# Three-dimensional, 2.5-minute, 7T phosphorus magnetic resonance spectroscopic imaging of the human heart using concentric rings

**DOI:** 10.1002/nbm.4813

**Published:** 2022-08-22

**Authors:** William T. Clarke, Lukas Hingerl, Bernhard Strasser, Wolfgang Bogner, Ladislav Valkovič, Christopher T. Rodgers

**Affiliations:** 1 Wellcome Centre for Integrative Neuroimaging, FMRIB, Nuffield Department of Clinical Neurosciences, University of Oxford, Oxford, UK; 2 High-field MR Centre, Department of Biomedical Imaging and Image-guided Therapy, Medical University of Vienna, Vienna, Austria; 3 Oxford Centre for Clinical Magnetic Resonance Research, Radcliffe Department of Medicine, University of Oxford, Oxford, UK; 4 Department of Imaging Methods, Institute of Measurement Science, Slovak Academy of Sciences, Bratislava, Slovakia; 5 Wolfson Brain Imaging Centre, Department of Clinical Neurosciences, University of Cambridge, Cambridge, UK

**Keywords:** ^31^P, CRT, heart, MRSI, phosphorus, spectroscopy

## Abstract

A three-dimensional (3D), density-weighted, concentric rings trajectory (CRT) magnetic resonance spectroscopic imaging (MRSI) sequence is implemented for cardiac phosphorus (^31^P)-MRS at 7 T. The point-by-point k-space sampling of traditional phase-encoded chemical shift imaging (CSI) sequences severely restricts the minimum scan time at higher spatial resolutions. Our proposed CRT sequence implements a stack of concentric rings, with a variable number of rings and planes spaced to optimise the density of k-space weighting. This creates flexibility in acquisition time, allowing acquisitions substantially faster than traditional phase-encoded CSI sequences, while retaining high signal-to-noise ratio (SNR). We first characterise the SNR and point-spread function of the CRT sequence in phantoms. We then evaluate it at five different acquisition times and spatial resolutions in the hearts of five healthy participants at 7 T. These different sequence durations are compared with existing published 3D acquisition-weighted CSI sequences with matched acquisition times and spatial resolutions. To minimise the effect of noise on the short acquisitions, low-rank denoising of the spatiotemporal data was also performed after acquisition. The proposed sequence measures 3D localised phosphocreatine to adenosine triphosphate (PCr/ATP) ratios of the human myocardium in 2.5 min, 2.6 times faster than the minimum scan time for acquisition-weighted phase-encoded CSI. Alternatively, in the same scan time, a 1.7-times smaller nominal voxel volume can be achieved. Low-rank denoising reduced the variance of measured PCr/ATP ratios by 11% across all protocols. The faster acquisitions permitted by 7-T CRT ^31^P-MRSI could make cardiac stress protocols or creatine kinase rate measurements (which involve repeated scans) more tolerable for patients without sacrificing spatial resolution.

## 1 Introduction

Phosphorus magnetic resonance spectroscopy (^31^P-MRS) allows measurement of the energy metabolism of the human heart in vivo, specifically the ratio of phosphocreatine to adenosine triphosphate (PCr/ATP), which is a biomarker of heart failure.^1^ To date, three-dimensional (3D) localised ^31^P-MRS measurements of the human heart have used chemical shift imaging (CSI) with Cartesian phase-encoded k-space sampling, single-voxel 3D image-selected in vivo spectroscopy (ISIS) or single-voxel STEAM pulse sequences.^2–4^ While CSI offers optimal signal-to-noise ratio (SNR) per unit time, the point-by-point sampling of k-space severely restricts the minimum scan time at higher spatial resolutions.^5^

Long acquisition times can restrict our ability to acquire data in a timeframe that is tolerable for clinical purposes. However, they are particularly restrictive when multiple, repeated acquisitions are needed for either stress protocols,^6^ or to noninvasively measure chemical kinetics of the oxidative phosphorylation system.^7,8^ These protocols, which are thought to provide more sensitive detection of underlying pathological processes,^9,10^ have typically been achieved by lowering spatial resolution, even reducing phase-encoded localisation to a single dimension,^11,12^ leading to significant partial volume effects.^13,14^ Large voxel volumes^7^ as well as cardiorespiratory motion are both responsible for partial volume effects, which are therefore apparent in protocols both with cardiac gating^15^ and without.^7^

Employing fast MRSI readout trajectories, it could be possible to leverage the approximately 2.8 times increase in SNR^3^ to achieve close to the theoretical 7.8-times (2.8^2^) speed increase when moving from 3 to 7 T, which is not feasible with point-by-point Cartesian sampling. Concentric rings trajectory (CRT)-MRSI is an attractive option because it has been shown to deliver high-resolution ^1^H-MRSI of the brain with close to optimal SNR-per-unit-time.^16,17^

Here, we propose a 3D density-weighted CRT-MRSI sequence to achieve fast ^31^P-MRSI of the human heart. This is achieved by modifying a previously implemented ^1^H-CRT-MRSI sequence^18^ to include full 3D density weighting to achieve a compact 3D point-spread function (PSF) in the acquisition, thereby avoiding the loss of SNR associated with postacquisition reweighting.^19^

MRSI acquisitions have highly redundant data, and are therefore particularly well suited to postprocessing with low-rank denoising^20,21^ to improve metabolite quantification precision. Here, low-rank denoising could mitigate the expected loss of SNR when reducing the sequence acquisition time. We therefore also compared the effects of an optimised low-rank denoising approach^21^ on data acquired using CSI and CRT trajectories.

In this work we demonstrate the feasibility of a 3D density-weighted CRT sequence for rapid ^31^P-MRSI of the human heart at 7 T. We compare this methodology with previously published sequences for reduced acquisition duration (with fixed resolution) or increased spatial resolution (with equivalent maximum scan time).^14^ In addition, we assess the impact of modern optimised low-rank denoising^21^ postprocessing on rapidly acquired CSI and CRT MRSI data. We aim ultimately to present a state-of-the-art approach to human cardiac metabolic imaging.

## 2 Methods

### 2.1 Sequence design

**Figure 1:**
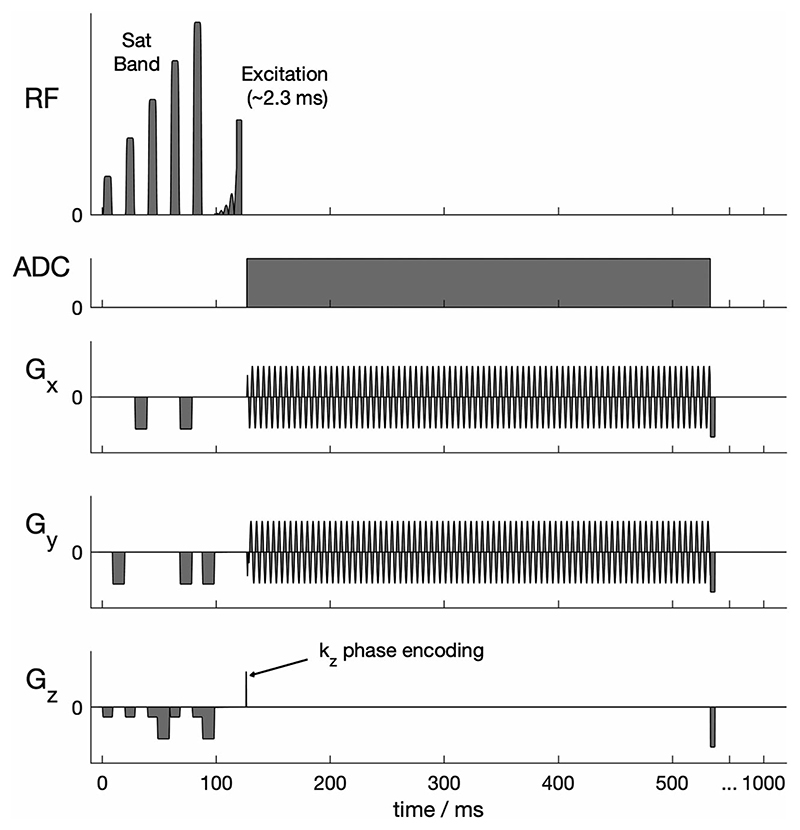
Pulse sequence diagram (single repetition time). Not to scale. The 3D concentric rings trajectory (CRT) sequence with density-weighted k-space acquisition is preceded by a B_1_-insensitive train to obliterate signal (BISTRO) saturation band module suitable for suppressing skeletal muscle signal at 7 T using surface coils. An asymmetric shaped excitation pulse providing minimal amplitude and phase variation over an ~ 2.5 kHz bandwidth was implemented (See Figure 4 of reference^3^ for pulse details). ADC, analogue to digital conversion; RF, radiofrequency

**Figure 2:**
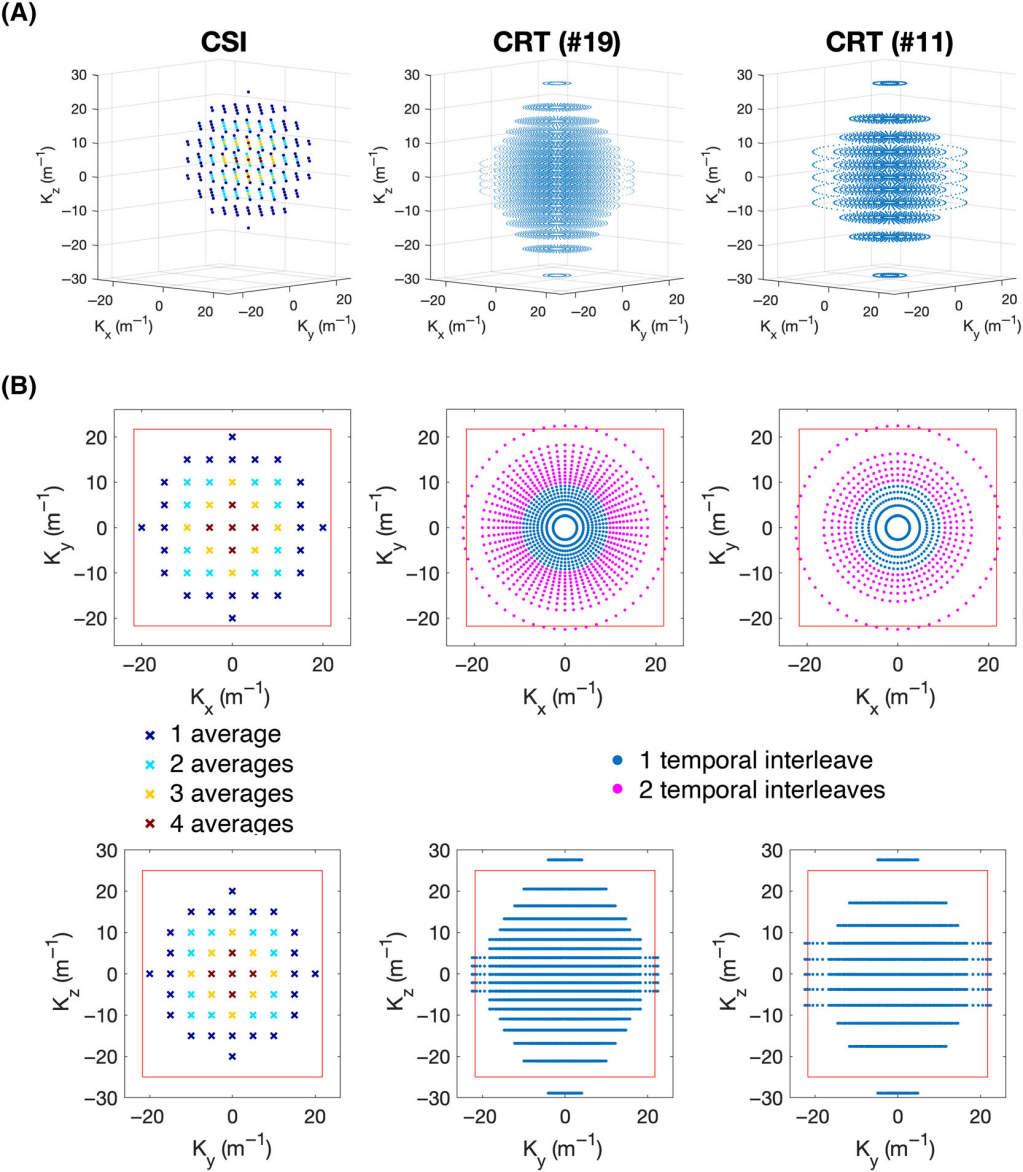
(A) 3D plots of the k-space trajectories of the Cartesian acquisition-weighted chemical shift imaging (CSI) (left), concentric rings trajectory (CRT) sequence with 19 rings/partitions (middle), and CRT sequence with 11 rings/partitions (right). At each k-space location marked a time domain signal is acquired (not shown). (B) Trajectories of the sequences shown in the xy-plane (top) and yz-plane (bottom). The red box marks the extent of the k-space (1/Δ_x/y_). The CSI acquisition weighting is illustrated via different colour-coding, with the central points sampled more frequently than the outer. The magenta points in the CRT sequence show rings acquired using two temporal interleaves to overcome spectral bandwidth limitations

A density-weighted 3D-CRT sequence was created by modifying a previously published equidistant ring 3D-CRT sequence.^18^ The sequence diagram of the modified sequence is shown in Figure 1. The MUSICAL^22^ coil-sensitivity scans were removed from the original sequence because they rely on the unsuppressed water signal that has no analogue in ^31^P-MRSI. The CRT readout gradients were modified with 3D density weighting (Figure 2). The density-weighting function *w*(*k*) was implemented as described in Equation (1). Note that Equation (1) also appears as Equation 4 in reference^19^ and as Equation (2 in reference.^23^)

(1) $$w(k)=\frac{\beta }{2}NA_{tot}\left(1+\text{cos}\left(2\pi \frac{k\text{Δ}x_{\text{nom}}}{\alpha }\right)\right),$$

where Δx_nom_ is the nominal spatial resolution, and *NA_tot_* is the total number of acquisitions, which for this work is set to one. *α* and *β* are constants set according to the Raleigh criterion, as described elsewhere.^19,23^ The weighting was implemented by placing rings with irregularly spaced radii (in the k_xy_-plane) on irregularly spaced planes (in the k_z_-direction, Figure 2). In this work, *α* was set to 1.71 in the k_xy_-plane and 1.61 in the k_z_-direction. *β* was set to 1.47 and 1.25, respectively. *α* and *β* values were chosen from literature values^19,23^ and simulation of the PSF for the CRT sequence trajectory with the 1D, 2D and 3D *a* and β values given in the literature. Density weighting in the k_xy_ plane (concentric rings) was implemented in the sequence using the process described in reference.^17^ For the k_z_ direction, the position of the planes was calculated similarly; plane positions were calculated by uniformly sampling along the cumulative distribution function of Equation (1). This was implemented by using a series expansion to numerically approximate the inverse cumulative distribution function. A detailed description of the implementation is provided in the supporting information.

To complement the density weighting, the number of rings in each plane was tuned to elliptically sample k-space in the k_z_-direction. Up to two temporal interleaves were used to achieve a fixed dwell time (spectral bandwidth) in rings that would otherwise have exceeded the hardware gradient slew rate limits. Total sequence duration was adjusted by varying the number of rings and k_z_ partitions, while keeping the maximum k-space coverage identical and thus the nominal resolution in the x, y and z directions equal.

To aid comparison with the established CSI sequence, we have implemented the same RF excitation pulse and outer volume saturation scheme as previously described.^3^ The excitation used an asymmetric 2.4-ms, shaped, constant phase pulse (Figure 1) designed to uniformly excite metabolites between –3 and 8 ppm (i.e., 2,3-diphosphoglycerate [2,3-DPG], phosphodiester [PDE], PCr and γ-ATP) when centred 270 Hz from PCr. A single ‘B_1_-insensitive train to obliterate signal’ (BISTRO) saturation band to suppress the chest wall signal was added to the sequence,^24^ and applied each repetition time.

### 2.2 Reconstruction

CSI data were reconstructed online using a modified version of the vendor’s reconstruction code.^3,25^ CRT data were reconstructed offline using the nonuniform fast Fourier transform (NUFFT) toolbox with min-max Kaiser-Bessel kernel interpolation and two-fold oversampling^26^ in MATLAB (MathWorks, Natick, MA, USA). Density compensation was not applied in addition to the trajectory density weighting. Individual coil data were combined using the whitened singular value decomposition (WSVD) algorithm.^25^

### 2.3 Simulation and phantom validation

The density-weighted CRT sequence was validated through simulations and phantom scans. The SNR and PSF of the sequence were characterised relative to an acquisition-weighted CSI sequence on a point-source phantom.^3^ The measured PSF was compared with the numerically simulated PSF.

CSI and CRT data with closely matched parameters were acquired on a phantom containing a 2 × 2 × 2 cm^3^ cube of 1 M K_2_HPO_4_ in a large tank filled with saline. Phantom data were acquired on a whole-body Siemens Magnetom 7-T scanner (Erlangen, Germany) equipped with a combined 10 cm ^1^H/15 cm ^31^P quadrature-pair transmit-receive surface coil.^27^ The acquisition grid was placed to centre a voxel over the point source. Seven different acquisitions were made with matched field of view (200 × 200 × 200 mm^3^), spectral bandwidth (8 kHz), T_R_ (1 s) and RF pulse voltages:

1. Acquisition-weighted CSI with 2 × 2 × 2 cm^3^ resolution, 10 × 10 × 10 matrix and N = 4 at k = 0 giving a TA of 6:31 min;
2. As #1 but with N = 1 at k = 0 (TA: 4:31 min);
3. CRT with 19, 15, 13 and 11 rings/partitions (TA: 6:55, 4:12, 3:12, and 2:18 mins) reconstructed to a 10 × 10 × 10 Nyquist matrix; and
4. CRT with 18 rings/partitions (6:27 mins) reconstructed to a Nyquist matrix size of 12 × 12 × 12.

The PSF was predicted by passing a uniform unitary synthetic signal (dimensions 128 × 128 × 128 voxels, matched field of view [FoV], matched positional shift, NUFFT Kaiser-Bessel kernel, two-times padded) through the NUFFT adjoint operation, as formulated for the gradient trajectory of the matched phantom data, that is, the predicted PSF was calculated using identical reconstruction parameters (excepting padding) as the phantom data, and as described in the Reconstruction subsection (2.2). The predicted SNR was calculated accounting for the total acquisition time and voxel volume and normalised to the SNR measured for the acquisition-weighted CSI acquisition to account for effects such as phantom T_1_ and coil sensitivity that are equal in all scans. Phantom-measured PSF and SNR were compared with predicted values.

### 2.4 In vivo comparison of CSI and CRT

Five healthy subjects (four males and one female; 74 ± 11 kg; age 30 ± 3 years) were scanned in a supine position using the same hardware as above (whole-body Siemens Magnetom 7-T scanner, 10 cm ^1^H/15 cm ^31^P quadrature-pair transmit-receive surface coil), with the coil positioned over the heart.^27^ Each subject was scanned using a range of CSI and CRT sequences with different acquisition times and sampling densities. The specific details of each sequence are given in Table 1. The protocols included a previously described CSI sequence^14^ (Protocol 1 in Table 1). Hereafter, we refer to each protocol as ‘${\text{METHOD}}_{\text{Resolution}}^{\text{Acquisition}\;\text{time}}$’, for example, ${\text{CSI}}_{10\times 10\times 10}^{6\;\min\;31\;s}$ is Protocol 2 in Table 1. Other protocol parameters were closely matched to those used for the previously described CSI sequence. Position, orientation, FoV, RF pulse voltages and repetition times of all protocols were matched. Readout bandwidth was dependent on acquisition method because the CRT spectral bandwidth is limited by hardware gradient slew rates. A maximum of two temporal interleaves was used so as not to prolong the acquisition time. This resulted in a CRT bandwidth less than CSI, which has an excessive bandwidth (8000 Hz) for the excited bandwidth (approximately 2500 Hz). Per subject B_0_ shimming was not carried out prior to ^31^P acquisitions. This decision was informed by previous research showing the limited effect of B_0_ shimming on ^31^P-MRSI data quality.^14^

Additionally, ^1^H CINE FLASH images with pulse oximeter gating were acquired in each subject for anatomical spectral localisation and volume of interest (VOI) identification. Images were acquired in four- and two-chamber long axis and three short axis views (apical, mid and basal). Only the images from midend diastole (phase 5–6 of 6/7), corresponding to a relatively stationary and long-lived cardiac phase, were used for VOI identification. From these structural images, anterior-, mid- and posterior interventricular septal voxels from apical, mid and basal short-axis views of the heart were manually picked based on our institution's standard anatomical landmarks. In addition, pure ventricular voxels (blood voxels) were selected from the right ventricle (RV) and left ventricle (LV) from apical, mid and basal short-axis views. Voxels were selected based on the 10 × 10 × 10 CSI grid with nearest neighbour interpolation used to select voxels from other resolutions. Selection was performed blinded to the MRS data.

### 2.5 Preprocessing and fitting

Data processing was carried out using the OXSA toolbox.^28^ Spectra were corrected for frequency offset and DC offset. Then peaks were fit with the advanced method for accurate, robust and efficient spectral fitting (AMARES).^28,29^ Prior knowledge specified 11 Lorentzian peaks (α, β, ATP multiplet components, PCr, PDE and the two peaks of 2,3-DPG), with fixed amplitude ratios and scalar couplings for each multiplet. Fitting was initialised with starting times measured from each sequence simulated in the vendor's simulation environment, corresponding to 0.35 and 0.85 ms for CSI and CRT, respectively. Blood contamination and partial saturation were corrected as previously described.^3,28^ Metabolite ratios and ratio uncertainties are reported for PCr/ATP.

To aid sequence comparison irrespective of intersubject differences in the PCr/ATP ratio, we also computed normalised PCr/ATP ratios according to:

(2) $$R_{\text{Normalised}}^{i,s}==R_{\text{Mean}}\times \left(\frac{R^{i,s}}{R_{{\text{CSI}}_{10\times 10\times 10}^{6\min\;31\;s}}^{i,s}}\right),$$

(3) $$R_{\text{Mean}}=\frac{1}{N}\sum_{s}\sum_{i}R_{{\text{CSI}}_{10\times 10\times 10}^{6\;\min\;31\;s}}^{i,s},$$

where *R^I,s^* is the saturation and blood-corrected PCr/ATP ratio of the *i*th voxel of the *s*th subject for a particular protocol. *R*_Mean_ scaled all values to the mean saturation and blood-corrected PCr/ATP value of the ${\text{CSI}}_{10\times 10\times 10}^{6\;\min\;31\;s}$ protocol.

### 2.6 Spatiotemporal denoising

Local (sliding-window) low-rank spatiotemporal denoising was applied to all reconstructed frequency domain MRSI data.^20,21^ Patch size was chosen to be 3 × 3 × 3 with a stride of one in all directions. Automatic rank selection was applied patch-wise using the Marchenko-Pastur distribution method.^21,30^ This formed a denoised representation of each reconstructed MRSI dataset. The denoising code is open-source and available online (https://git.fmrib.ox.ac.uk/wclarke/low-rank-denoising-tools), and as an installable package ‘mrs_denoising_tools’ via the package managers PyPi (Python Software Foundation, Wilmington, DE, USA) and Conda (Anaconda Inc, Austin, TX, USA). The denoised data were also fitted and corrected for blood contamination and partial saturation, following the same procedure described above.

### 2.7 Sequence comparison

Results were compared with the reference ${\text{CSI}}_{10\times 10\times 10}^{6\;\min\;31\;s}$ dataset using saturation and blood-corrected PCr/ATP ratios in the selected voxels using the Wilcoxon signed rank test (for paired measurements). The comparison was repeated for denoised results, while still comparing with the nondenoised reference ${\text{CSI}}_{10\times 10\times 10}^{6\;\min\;31\;s}$ dataset.

## 3 Results

### 3.1 Simulation and phantom validation

**Figure 3:**
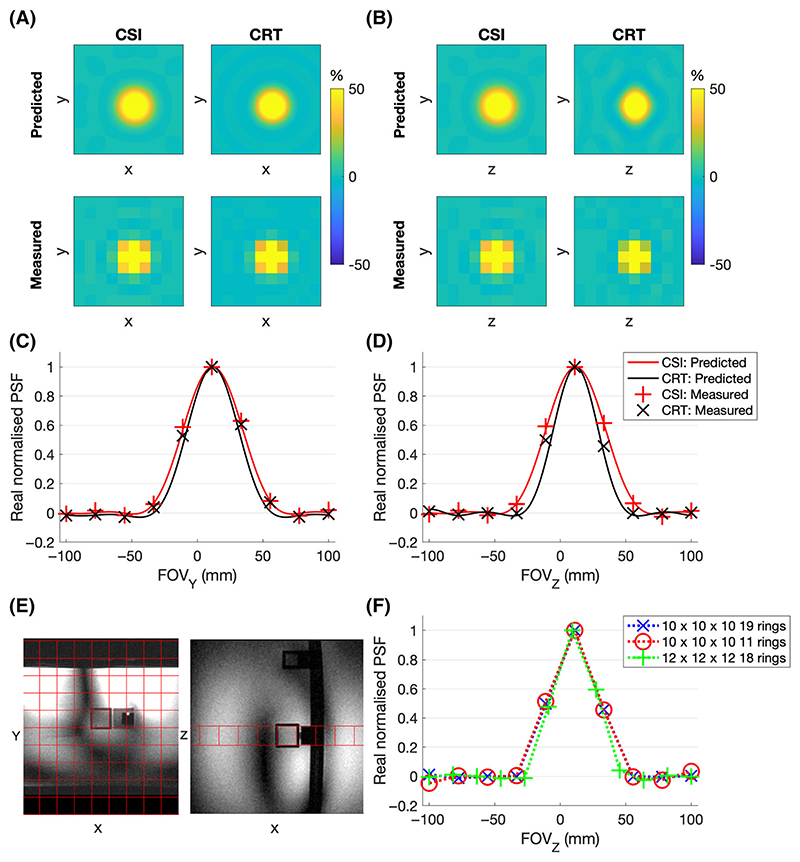
Predicted and measured point-spread functions (PSFs) of the chemical shift imaging (CSI) and 19 concentric rings trajectory (CRT) sequence in the (A) XY-plane and (B) YZ-plane. The PSF profile on the y and z axis is plotted through the central point in (C) and (D). (E) Pointsource phantom used to measure the PSF with the 10 × 10 × 10 spatial grid overlaid. ^31^P signal only arises from the central cube. (F) Effect of varying resolution and number of rings on the measured PSF in the z-direction. FOV, field of view

Phantom experiments showed that the measured CRT PSF matched the predicted low ripple PSF both in the plane of the rings (Figure 3A,C) and through the plane in the third dimension (Figure 3B,D). The z-direction PSF of the CRT was narrower than the CSI potentially indicating a small deviation from the desired weighting function. Reducing the number of rings to 10 from 19 did not cause deterioration of the central lobe of the PSF (full width at half maximum [FWHM] increased by 0.2%; Figure 3F). The high-resolution, 18-ring ${\text{CRT}}_{12\times 12\times 12}^{6\;\min27\;\text{s}}$ sequence had similar PSF with low ripple. The FWHM of the central lobe was 10% smaller than the 10 × 10 × 10 sequence, less than predicted by the resolution increase, but explained by the presence of signal arising outside the smaller voxel.

**Figure 4:**
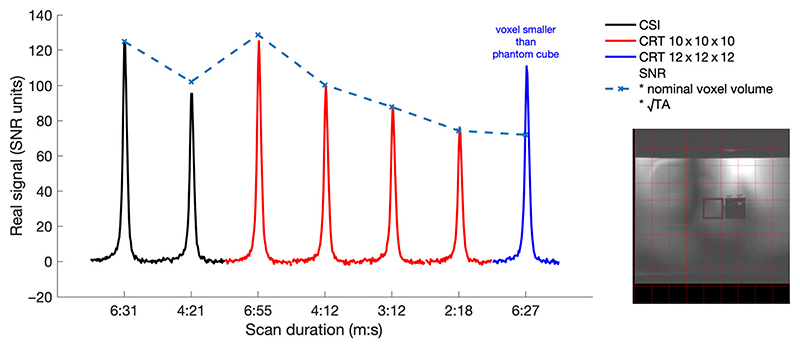
Measured signal (scaled to signal-to-noise ratio [SNR] units) from the point-source phantom acquired with chemical shift imaging (CSI) and concentric rings trajectory (CRT) sequences of varying acquisition times. The predicted SNR is calculated relative to the ${\text{CSI}}_{10\times 10\times 10}^{6\;\min31\;\text{s}}$ scan, adjusting for the nominal voxel volume and acquisition time of each protocol. The deviation for the short${\text{CSI}}_{10\times 10\times 10}^{4\;\min\;21\;\text{s}}$ scan arises because CSI can only sample integer numbers of points at each k-space location, thus SNR is lost in postacquisition reweighting. The larger deviation for the higher resolution CRT scan (6 min 27 s) has a voxel smaller than the ‘point-source’, resulting in signal bleed. The image shows the position of the 10 × 10 imaging matrix (red) over the phantom ‘point-source’. ^31^P signal only arises from the central cube

SNR performance mostly showed the predicted relationship, dependent on acquisition time (Figure 4). SNR was matched between time-matched acquisition-weighted CSI and density-weighted CRT sequences. The postacquisition reweighting of the single average (uniform weighted) CSI results in an SNR loss. Therefore the ${\text{CSI}}_{10\times 10\times 10}^{4\;\min\;21\;\text{s}}$ produced lower SNR than predicted, and lower SNR than the density-weighted ${\text{CSI}}_{10\times 10\times 10}^{4\;\min\;21\;\text{s}}$ scan. The high-resolution ${\text{CRT}}_{12\times 12\times 12}^{6\;\min\;27\;\text{s}}$ produced higher SNR than predicted, but this is likely to arise from the signal outside the measured voxel bleeding into the measured voxel because of the PSF.

### 3.2 In vivo results

**Figure 5:**
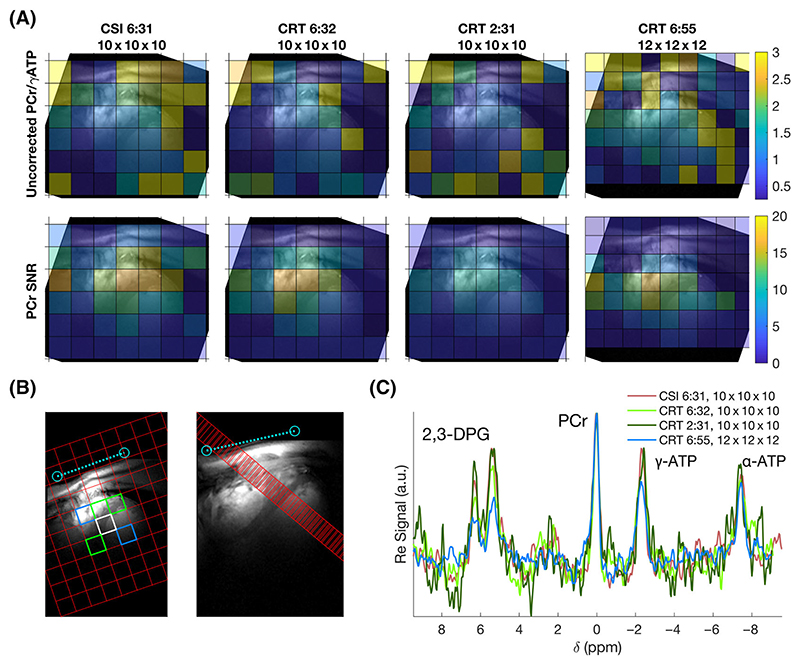
(A) Uncorrected PCr/γ-ATP ratio and PCr SNR maps of a midshort-axis slice for four of the tested sequences overlaid on shortaxis localiser images. Maps from all protocols are shown in Figure S1. (B) Target voxel locations overlaid on short-axis and four-chamber localiser images. Green + white = septum, blue = right and left ventricle blood-pools. The intersection of one of the ^31^P coil elements with the proton image is marked in light blue. (C) Spectra from a single subject’s midseptal voxel (white in (B)) for four of the tested sequences. Line colours correspond to Table 1. Spectra in (C) have been apodised using a 40 Hz exponential filter

^31^P-MRSI was acquired successfully using 3D density-weighted CRT with acquisition times down to 2 min 31 s in all five subjects. Example PCr/ATP and PCr SNR maps of the midshort-axis slice from four sequences in one subject are shown in Figure 5A. Maps for all sequences are shown in Figure S1. The selection of voxels based on standard anatomical landmarks to include only cardiac and surrounding voxels resulted in 52 septal myocardial voxels selected across five subjects (10–11 per subject; three apical, three or four mid and four basal) and 10 ventricular voxels (two per subject). Example manually selected interventricular septal and ventricular voxels for analysis are shown in Figure 5B. Spectra from midseptal voxels of the same four sequences and subject are shown in Figure 5C. Spectra from a whole slice of myocardial voxels are shown in Figure S2.

Results of the in vivo comparison are summarised in Table 1. The matched filter PCr peak SNR followed the expected relationship decreasing in line with total acquisition time and voxel volume. The 10 × 10 × 10 isotropic resolution results in a nominal voxel volume of 11.5 ml and the high resolution (12 × 12 × 12) in a nominal volume of 6.7 ml. PCr SNR was 7.3 or greater for the shortest ${\text{CRT}}_{10\times 10\times 10}^{2\;\min\;31\;\text{s}}$ sequence, with a mean (±SD) of 8.8 ±6.0. A higher SNR (9.2 ±6.8) was measured for the high-resolution ${\text{CRT}}_{12\times 12\times 12}^{6\min55\text{s}}$ sequence. SNR-per-unit time was calculated, and showed that CSI was the most SNR optimum, but CRT achieved between 98% and 86% of the SNR of CSI (with the same voxel size). More than 73% of septal voxels could be quantified with a relative PCr/ATP Cramér-Rao lower bound (CRLB) less than 25% for all sequences. Over 82% of voxels could be quantified for all sequences with a relative PCr/ATP CRLB less than 30%.

**Figure 6:**
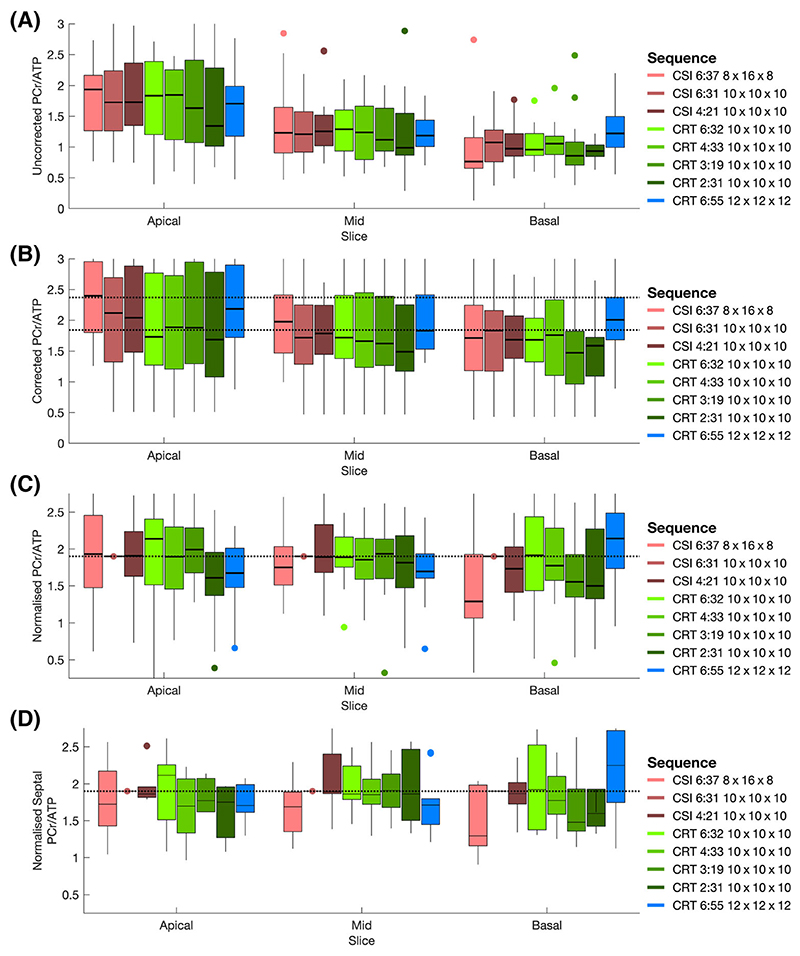
(A) Measured PCr/ATP ratios from all five subjects in target voxels (Figure 5B) from apical, mid and basal short-axis slices in each tested sequence variant. (B) Saturation and blood-corrected PCr/ATP ratios from all five subjects in target voxels (Figure 5B) from apical, mid and basal short-axis slices in each tested sequence variant. Dashed lines show the range of measured corrected PCr/ATP ratios from Ellis et al.^14^ (C) PCr/ATP ratios normalised per voxel to the values of the ${\text{CSI}}_{10\times 10\times 10}^{6\;\min\;31\;\text{s}}$ sequence. The dashed line shows normalised value scaled to mean septal value. (D) PCr/ATP ratios of just the septal voxels (green + white; Figure 5B) normalised to the values of the ${\text{CSI}}_{10\times 10\times 10}^{6\;\text{min}\;31\;\text{s}}$ sequence. The dashed line shows the normalised value

Table 1 and Figure 6 summarise the range of PCr/ATP ratio values measured in this study. A clear dependence on slice (apical, mid, basal) is observed for uncorrected PCr/ATP values, ranging from approximately two (apical) to one (basal). Saturation and blood correction (Figure 6B) reduce this dependence, although it remains.

Figure 6C,D show values of PCr/ATP normalised to the per-voxel value measured by the ${\text{CSI}}_{10\times 10\times 1}^{6\;\min\;31\;\text{s}}$ sequence, and then scaled to the mean septal PCr/ATP value. Median values measured by CRT sequences were close to the CSI values in the midslice (Figure 6C) and midslice septal (Figure 6D) voxels. Variance is seen to increase as acquisition time decreases. Values are less consistent in apical and basal slices. Different through plane resolutions ($CSI_{8\times 16\times 8}^{6\;\min\;37\;\text{s}}$ and ${\text{CRT}}_{12\times 12\times 1}^{6\;\min\;55\;\text{s}}$) produce notably different results despite the application of blood and saturation correction.

The statistical analysis of corrected PCr/ATP values indicated that only the high-resolution $CRT_{12\times 12\times 12}^{6\;\min\;55\;\text{s}}$ scan measured significantly different *(p* < 0.05, Wilcoxon signed rank test) PCr/ATP distributions from the reference CSI scan.

Spectra arising from voxels identified as ‘blood-pool’ (i.e., LV and RV) are displayed in Figure S3. Both groups of blood-pool voxels showed substantial PCr signal in all sequences, with average DPG/PCr ratios of 0.79 ± 0.3 (LV) and 1.13 ± 0.6 (RV), compared with 0.71 ± 0.3 for the midinterventricular septum. Across all subjects and sequences the mean uncorrected PCr/ATP ratio was lower in both blood-pool voxels, 1.02 (LV) and 0.95 (RV), compared with 1.19 for the midinterventricular septum.

### 3.3 Denoised CRT results

**Figure 7:**
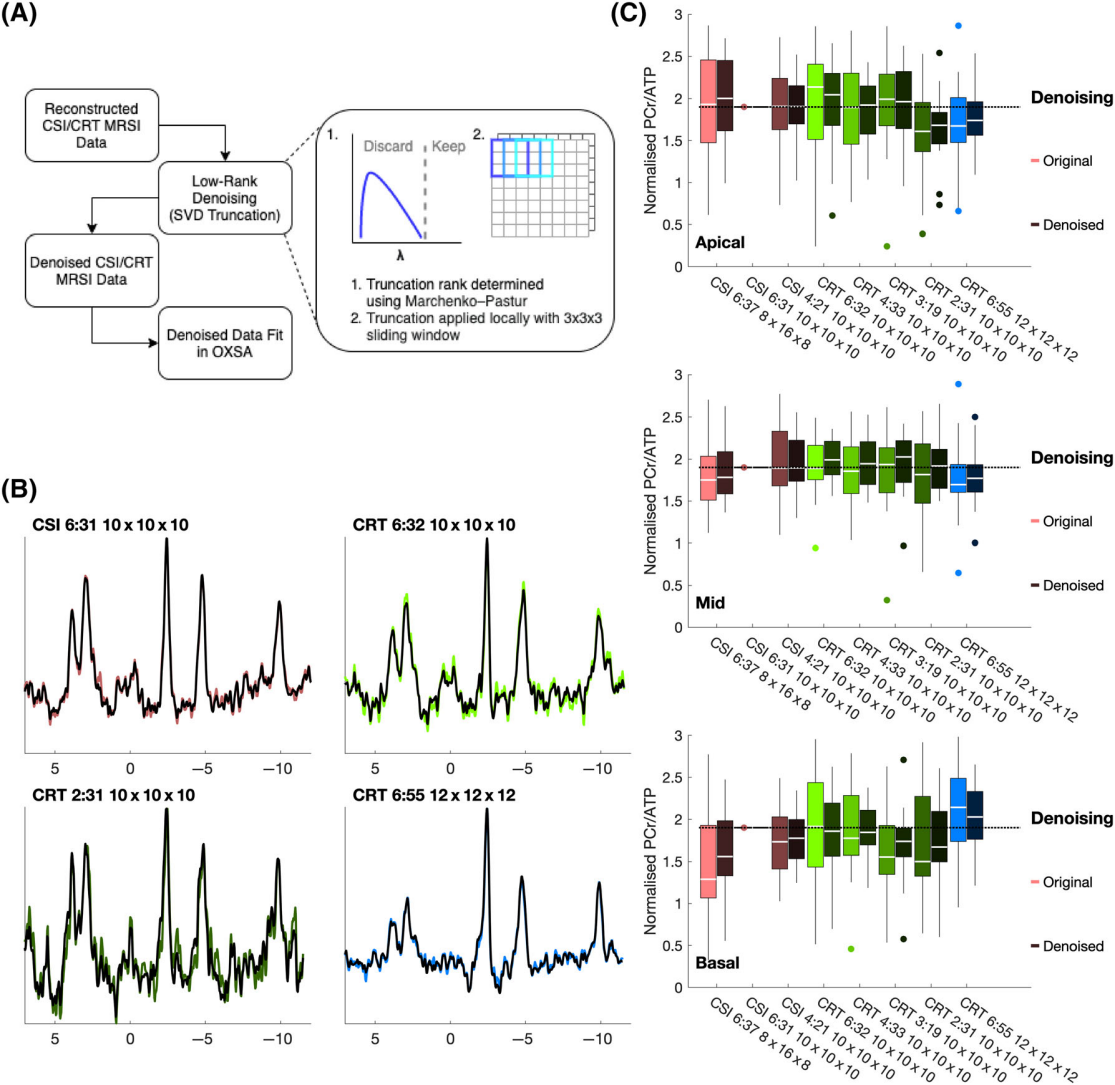
Low-rank denoising. (A) Denoising process. Reconstructed noisy chemical shift imaging (CSI) and concentric rings trajectory (CRT) undergo low-rank denoising before being refit in OXSA. Automatic rank selection is used to truncate overlapping 3 x 3 x 3 patches of MRSI data, with the result being the average of the overlapping patches. (B) Denoised (dark) midseptal spectra overlaid on original noisy data (light) from one subject and four scans (isotropic CSI, long and short CRT, and high-resolution CRT). (C) Normalised PCr/ATP ratios of original (noisy, light colour) and denoised (dark colour) voxels in apical, mid and basal slices. SVD, singular value decomposition

All low-rank denoised spectra showed apparent denoising (Figure 7B). Across all protocols, measured SNR, ignoring the effect of nonuniform variance, was 2.2 times higher than the original ‘noisy’ spectra. However, there was substantial variance between subjects in each protocol and between protocols, as shown by the large SD reported in Table 1. Denoised and corrected PCr/ATP values are reported in Table 1, and show similar mean values, although only slightly reduced standard deviations, which is driven by interslice range. However, measured PCr/ATP ratios normalised to isotropic CSI measured PCr/ATP (Figure 7C) showed reduced variance, particularly for the intermediate duration CRT protocols. Across all denoised CRT protocols, mean denoised RMSE was 89% ± 8% of the original noisy RMSE (compared with the values of the reference ${\text{CSI}}_{10\times 10\times 10}^{6\;\min\;31\;\text{s}}$ sequence).

Only the denoised ${\text{CSI}}_{12\times 12\times 12}^{6\min55\;\text{s}}$ protocol was found to have a statistically different (*p* < 0.05, Wilcoxon signed rank test) blood and saturation-corrected PCr/ATP distribution from the reference, not denoised ${\text{CSI}}_{10\times 10\times 10}^{6\;\min\;31\;\text{s}}$.

## 4 Discussion

Density-weighted 3D CRT MRSI has been demonstrated for fast 3D-localised cardiac ^31^P-MRS at 7 T. Phantom measurements showed no loss of SNR compared with SNR optimal CSI encoding.^5^ In vivo PCr/ATP maps are consistent with maps generated from previously published 3D phase-encoded CSI sequences. Specifically, PCr/ATP values in the interventricular septum, a common region of interest, have been found to be comparable with previously published CSI sequences.

The use of a 3D density-weighted CRT sequence allows for flexibility in the acquisition time of a ^31^P-MRSI sequence, offering the ability to acquire rapid measurements without degradation of the PSF or further loss of SNR at a chosen resolution and T_R_. Although CSI is SNR optimal compared with fast MRSI,^5^ SNR-per-unit-time was higher for CRT (#5) than the equivalent time, postacquisition reweighted CSI scan (1-average, #3). SNR was calculated using a matched filter, which mitigates the effects of differing bandwidths on the comparison, with the receiver bandwidth of CRT (177 kHz) higher than that of CSI (16 kHz).

In this study, a ${\text{CRT}}_{10\times 10\times 10}^{2\;\min\;31\;\text{s}}$ acquisition was shown to produce good quality results not significantly different from a conventional ${\text{CSI}}_{10\times 10\times 10}^{6\;\min\;31\;\text{s}}$ scan of identical resolution. This demonstrates a 2.63- and 1.73-fold reduction in scan time compared with that previously described^14^ and the shortest feasible Cartesian-sampled CSI sequence at matched T_R_ ${\text{CSI}}_{10\times 10\times 10}^{4\;\min\;21\;\text{s}}$. In turn, this allows for several acquisitions to be performed within a scan session, for instance during an exercise or pharmacological stress intervention (with subsequent recovery) for dynamic information about the cardiac energetics. This would also be particularly desirable for creatine kinase flux measurements, which are currently extremely long. At both 3 and 7 T, protocols of four^11^ and six scans,^7^ respectively, have been suggested, with scan times of 84 and 82 min. A three-scan protocol has also been proposed for 3 T taking approximately 70 min.^12^ While the saturation transfer methods lower PCr SNR, so the same speed-ups might not be possible, use of CRT could reduce scan times from over 1 h to the 30–40-min region. One further use might be to decrease the time required for 3D-resolved acquisition of cardiac Pi utilising adiabatic excitation and long TR at 7 T.^31^

The implementation of the CRT sequence allows greater flexibility in the trade-off between spatial resolution and acquisition time than CSI. In this work we demonstrated a higher resolution sequence ${\text{CSI}}_{12\times 12\times 12}^{6\;\min\;55\;\text{s}}$ with a 6.6-ml voxel. This was acquired in 6 min 55 s, which is shorter than the minimum time predicted for a single average CSI sequence (8 min 35 s) of the same resolution. The longer CSI sequence would also suffer from SNR loss due to postacquisition reweighting if the same PSF was desired. In this work, no density compensation was applied in the CRT sequence reconstruction, however, it is likely the acquired trajectory deviates slightly from the desired density-weighting function, therefore compensation might yield small improvements in PSF and SNR.

The PCr/ATP ratios measured in this study are in good agreement with those measured by Ellis et al.,^14^ falling well within the standard deviation measured by the previous study. While the same CSI sequence, spectral processing, fitting and saturation and blood signal contamination correction was applied here, different RF coil hardware was used. Hence, the small differences in measured PCr/ATP between the studies probably reflect the different coil transmit profiles combined with imperfect saturation correction applied. This could also explain the differences in PCr/ATP ratios measured for different slices (apical, mid and basal) in this study. This does not interfere with the comparison of the acquisition schemes, as the same correction was applied across all variants. In this work no reproducibility metrics have been provided as repeated acquisitions on the same subjects were not made. Before the proposed sequence is extensively used a thorough assessment of scan-rescan reproducibility should be carried out and compared with previous CSI data.^14^

Resolution was found to have a strong effect on the measured PCr/ATP ratio. Both sequences with higher ${\text{CSI}}_{12\times 12\times 12}^{6\;\min\;55\;\text{s}}$ and lower ${\text{CSL}}_{8\times 16\times 8}^{6\;\min\;37\;\text{s}}$ through plane resolution than the 10 × 10 × 10 isotropic resolution measured different PCr/ATP ratio distributions. The direction of the change depended on the location of the voxel being compared. This is despite standard literature saturation and blood signal contamination corrections being applied. As is evident in Figure 5C, the high-resolution CRT has much lower 2,3-DPG signal in the septal voxels than the lower resolution datasets, yet blood correction does not fully account for the lower PCr/ATP measured in septal voxels of apical and mid slices. It is likely that the differing PSFs interact with the high (skeletal muscle) and low (blood or liver) PCr/ATP compartments to produce these differences. Spectra from blood-pool voxels show substantial PCr signal, likely because of partial volume in the PSF-broadened voxels combined with no motion compensation being employed in this study.

For this study, which focuses on an initial characterisation of the proposed methods compared with an existing acquisition-weighted CSI, a fixed TR (of 1 s) with no cardiac or respiratory gating was chosen to minimise per subject variation. Cardiac gating introduces subject-dependent TR (as a function of the individual's heart rate); this in turn affects:

1. partial saturation of magnetization; and
2. specific absorption rate, which in turn affects the available transmit power and efficacy of saturation of contaminating skeletal muscle signal.

Although the former is mitigated by saturation correction, the correction is incomplete due to average literature values of T_1_ and Biot-Savart-calculated flip angle maps.^3^

Nevertheless, the increased flexibility of the sequence acquisition timings and kt-space acquisition speed may permit development-integrated motion-correction methods into the sequence. Consistent acquisition within the end systolic phase through gating has shown improved reproducibility and SNR.^32^ Implementation on hardware permitting interleaved ^1^H navigator images would allow tracking of the myocardial position through all cardiorespiratory phases.^33^ Further dedicated study is needed to assess the effect of different motion-correction strategies, given the subtle findings to date.

Low-rank denoising produced a substantial denoising effect, as shown by reduced variance of PCr/ATP and lower RMSE compared with the ‘gold standard’ CSI sequence. Thus low-rank denoising has the potential to substantially mitigate the loss of SNR resulting from faster acquisitions. However, denoising will necessarily bias the measured PCr/ATP ratio and leads to nonuniform signal-dependent variance,^21^ so care must be taken in its use. For use in dynamic protocols with repeated sequence acquisitions, it might be possible to employ strategies to ensure similar denoising performance on each datapoint, such as rank estimation on the whole dynamic dataset.

Using CRT has some potential limitations compared with CSI. In non-Cartesian trajectories, like CRT, off-resonance will cause minor spatially dependent blurring of the PSF, which does not occur in CSI. The maximum expected off-resonance across the myocardium ±100 Hz^34^ combined with the short dwell time (transit time per concentric ring), will only produce small phase discontinuities in k-space (approx.. 0.1 cycles per ring), resulting in small amounts of blurring.^35^ Aliasing in CRT sequences is incoherent. Therefore, in CRT datasets, aliased signal could subtly influence measured metabolite ratios in myocardial voxels without obvious visual artefacts (as is the case in CSI). This is important in the case of cardiac ^31^P-MRS, where potentially contaminating tissues (skeletal muscle and liver) that contain the same metabolites at different concentrations are in close proximity. Care must also be taken with the increased susceptibility to off-isocentre distortions caused by gradient nonlinearity and strong spatial blurring associated with aliased spectral peaks, if the chosen spectral bandwidth is too narrow.^36^ We overcame the limits on spectral bandwidth by using relatively narrow-band excitation. We are not aware of aliasing in our reconstructions.

## 5 Conclusion

In this work we introduce a 3D density-weighted CRT sequence for rapid acquisition of ^31^P-MRSI in the human heart. The sequence is implemented on a whole-body Siemens 7-T scanner. The proposed sequence can measure the PCr/ATP ratio in the human septal myocardium in 2.5 min, which is 2.63 times faster than a standard CSI sequence with the same nominal voxel size of 11.5 ml. CRT can acquire high-resolution data (6.7-ml voxel volume) in only 6 min 55 s versus the minimal 8 min 35 s predicted for a single average weighted CSI, while retaining equal SNR. Low-rank denoising is particularly beneficial at these short scan times.

## Supplementary Material

Supporting Information

## Figures and Tables

**Table 1:** Top: protocol parameters. Colour coding is matched to subsequent figures. Bottom: results. Matched-filter PCr SNR, SNR normalised to the square root of unit time, mean (±SD) uncorrected PCr/ATP, saturation-corrected PCr/ATP, and number of voxels fitted with PCr/ATP CRLB < 30% from the septal voxels (total 52, 10–11 voxels per subject). Mean and SD is calculated over all septal voxels of all subjects. The table also shows the matched-filter PCr SNR and saturation-corrected PCr/ATP values from the same voxels after denoising. ‘SNR ratio’ is the ratio of ‘denoised PCr SNR’ to ‘PCr SNR’

Protocol #	1	2	3	4	5	6	7	8
***Acqusition parameters***							
**Trajectory**	**CSI**	**CSI**	**CSI**	**CRT**	**CRT**	**CRT**	**CRT**	**CRT**
**TA (m:ss)**	6:37	6:31	4:21	6:32	4:33	3:19	2:31	6:55
**NA (k=O)**	4	4	1					
**# rings/partitions**				20	16	14	12	19
**Reconstruction matrix**	8x16x8	10x10x10	10x10x10	10x10x10	10x10x10	10x10x10	10x10x10	12x12x12
**Nominal voxel (ml)**	11.3	11.5	11.5	11.5	11.5	11.5	11.5	6.7
**FoV (mm^3^)**	240*240*200	240*240*200	240*240*200	240*240*200	240*240*200	240*240*200	240*240*200	240*240*200
**Tr(s)**	1	1	1	1	1	1	1	1
**Bandwidth (Hz)**	8000	8000	8000	2778	2778	2778	2778	2778
**Time samples**	2048	2048	2048	720	720	720	720	720
**TA reduction compared to#l**	1.00	1.02	1.52	1.01	1.45	1.99	2.63	0.96
***Results***							
**PCrSNR**	14.5 (±10.9)	15.2 (±8.8)	11.2 (±7.4)	14.9 (±10.6)	11.4 (±7.2)	9.9 (±7.3)	8.8 (±6.0)	9.2 (±6.8)
**PCrSNR/✓T(%of max)**	5.6 (95%)	6.0 (100%)	5.3 (88%)	5.8 (98%)	5.4 (90%)	5.4 (91%)	5.5 (93%)	3.5 (59%)
**PCr/ATP**	1.07 (+0.63)	1.21 (±0.52)	1.37 (±0.59)	1.22 (±0.56)	1.19 (±0.58)	1.14 (±0.87)	1.03 (+0.71)	1.31 (±0.53)
**Corrected PCr/ATP**	1.85 (±0.89)	1.90 (±0.93)	1.82 (±0.97)	1.71 (±0.96)	1.80 (±1.02)	1.59 (±0.93)	1.52 (±0.97)	2.12 (±0.79)
**Septal voxels (#/52)**							
**CRLB <30%**	49	52	48	49	47	43	43	45
**CRLB <25%**	47	50	47	48	44	40	38	39
**Denoised PCr SNR**	31.3 (±15.6)	40.4 (±15.5)	24.6 (±9.9)	26.8 (±12.6)	20.8 (±9.2)	21.7 (±10.7)	19.7 (±9.3)	20.8 (±10.2)
**SNR Ratio**	2.2	2.7	2.1	1.8	1.9	2.2	2.2	2.3
**Denoised Corrected PCr/ATP**	1.80 (±0.81)	2.01 (±0.74)	1.91 (±0.79)	1.94 (±0.89)	1.93 (±0.87)	1.95 (±0.95)	1.57 (±0.95)	2.08 (±0.95)

Abbreviations: CRLB, Cramér-Rao lower bound; PCr/ATP, ratio of phosphocreatine to adenosine triphosphate; SNR, signal-to-noise ratio.

## Abbreviations used

2,3-DPG: 2,3-diphosphoglycerate
^31^P-MRS: phosphorus magnetic resonance spectroscopy
AMARES: advanced method for accurate, robust and efficient spectral fitting
ATP: adenosine triphosphate
BISTRO: B_1_-insensitive train to obliterate signal
CRT: concentric rings traj’ectory
CSI: chemical shift imaging
FLASH: fast low angle shot
FWHM: full width at half maximum
ISIS: image-selected in vivo spectroscopy
MUSICAL: multichannel spectroscopic data combined by matching image calibration data
MRSI: magnetic resonance spectroscopic imaging
NUFFT: nonuniform fast Fourier transform
PCr: phosphocreatine
PCr/ATP: ratio of phosphocreatine to adenosine triphosphate
PDE: phosphodiester
PSF: point-spread function
SNR: signal-to-noise ratio
WSVD: whitened singular value decomposition.

## References

[1] Neubauer S (2007). The failing heart — an engine out of fuel. N Engl J Med.

[2] Lamb HJ, Doornbos J, den Hollander JA (1996). Reproducibility of human cardiac 31P-NMR spectroscopy. NMR Biomed.

[3] Rodgers CT, Clarke WT, Snyder C, Vaughan JT, Neubauer S, Robson MD (2014). Human cardiac 31P magnetic resonance spectroscopy at 7 Tesla. Magn Reson Med.

[4] Apps A, Valkovič L, Peterzan M (2021). Quantifying the effect of dobutamine stress on myocardial Pi and pH in healthy volunteers: A 31P MRS study at 7T. Magn Reson Med.

[5] Pohmann R, von Kienlin M, Haase A (1997). Theoretical evaluation and comparison of fast chemical shift imaging methods. J Magn Reson.

[6] Levelt E, Rodgers CT, Clarke WT (2016). Cardiac energetics, oxygenation, and perfusion during increased workload in patients with type 2 diabetes mellitus. Eur Heart J.

[7] Clarke WT, Robson MD, Neubauer S, Rodgers CT (2017). Creatine kinase rate constant in the human heart measured with 3D-localization at 7 Tesla. Magn Reson Med.

[8] Bottomley PA, Ouwerkerk R, Lee RF, Weiss RG (2002). Four-angle saturation transfer (FAST) method for measuring creatine kinase reaction rates in vivo. Magn Reson Med.

[9] Weiss RG, Gerstenblith G, Bottomley PA (2005). ATP flux through creatine kinase in the normal, stressed, and failing human heart. PNAS.

[10] Butterworth EJ, Evanochko WT, Pohost GM (2000). The 31P-NMR stress test: an approach for detecting myocardial ischemia. Ann Biomed Eng.

[11] Schär M, El-Sharkawy A-MM, Weiss RG, Bottomley PA (2010). Triple repetition time saturation transfer (TRiST) 31P spectroscopy for measuring human creatine kinase reaction kinetics. Magn Reson Med.

[12] Schär M, Gabr RE, El-Sharkawy A-MM, Steinberg A, Bottomley PA, Weiss RG (2015). Two repetition time saturation transfer (TwiST) with spill-over correction to measure creatine kinase reaction rates in human hearts. J Cardiovasc Magn Reson.

[13] Dass S, Cochlin LE, Holloway CJ (2010). Development and validation of a short 31P cardiac magnetic resonance spectroscopy protocol. J Cardiovasc Magn Reson.

[14] Ellis J, Valkovič L, Purvis LAB, Clarke WT, Rodgers CT (2019). Reproducibility of human cardiac phosphorus MRS (31P-MRS) at 7 T. NMR Biomed.

[15] Clarke WT, Peterzan MA, Rayner JJ (2019). Localized rest and stress human cardiac creatine kinase reaction kinetics at 3 T. NMR Biomed.

[16] Chiew M, Jiang W, Burns B (2018). Density-weighted concentric rings k-space trajectory for 1H magnetic resonance spectroscopic imaging at 7 T. NMR Biomed.

[17] Hingerl L, Bogner W, Moser P (2018). Density-weighted concentric circle trajectories for high resolution brain magnetic resonance spectroscopic imaging at 7T. Magn Reson Med.

[18] Hingerl L, Strasser B, Moser P (2020). Clinical high-resolution 3D-MR spectroscopic imaging of the human brain at 7 T. Invest Radiol.

[19] Greiser A, von Kienlin M (2003). Efficient k-space sampling by density-weighted phase-encoding. Magn Reson Med.

[20] Nguyen HM, Peng X, Do MN, Liang Z (2013). Denoising MR spectroscopic imaging data with low-rank approximations. IEEE Trans Biomed Eng.

[21] Clarke WT, Chiew M (2022). Uncertainty in denoising of MRSI using low-rank methods. Magn Reson Med.

[22] Strasser B, Chmelik M, Robinson SD (2013). Coil combination of multichannel MRSI data at 7 T: MUSICAL. NMR Biomed.

[23] Pohmann R, von Kienlin M (2001). Accurate phosphorus metabolite images of the human heart by 3D acquisition-weighted CSI. Magn Reson Med.

[24] Luo Y, de Graaf RA, DelaBarre L, Tannús A, Garwood M (2001). BISTRO: An outer-volume suppression method that tolerates RF field inhomogeneity. Magn Reson Med.

[25] Rodgers CT, Robson MD (2016). Coil combination for receive array spectroscopy: Are data-driven methods superior to methods using computed field maps?. Magn Reson Med.

[26] Fessler JA, Sutton BP (2003). Nonuniform fast Fourier transforms using min-max interpolation. IEEE Trans Signal Process.

[27] Schaller B, Paritmongkol W, Magill AW, Robson M, Rodgers CT (2016). Proc Intl Soc Mag Reson Med.

[28] Purvis LAB, Clarke WT, Biasiolli L, Valkovič L, Robson MD, Rodgers CT (2017). OXSA: An open-source magnetic resonance spectroscopy analysis toolbox in MATLAB. PLoS ONE.

[29] Vanhamme L, van den Boogaart A, Van Huffel S (1997). Improved method for accurate and efficient quantification of MRS data with use of prior knowledge. J Magn Reson.

[30] Veraart J, Novikov DS, Christiaens D, Ades-aron B, Sijbers J, Fieremans E (2016). Denoising of diffusion MRI using random matrix theory. NeuroImage.

[31] Valkovič L, Clarke WT, Schmid AI (2019). Measuring inorganic phosphate and intracellular pH in the healthy and hypertrophic cardiomyopathy hearts by in vivo 7T 31P-cardiovascular magnetic resonance spectroscopy. J Cardiovasc Magn Reson.

[32] Wampl S, Körner T, Valkovič L (2021). Investigating the effect of trigger delay on cardiac 31P MRS signals. Sci Rep.

[33] Wampl S, Körner T, Roat S (2020). Proc Intl Soc Mag Reson Med.

[34] Tao Y, Hess AT, Keith GA (2015). Optimized saturation pulse train for human first-pass myocardial perfusion imaging at 7T. Magn Reson Med.

[35] Wu HH, Lee JH, Nishimura DG (2008). MRI using a concentric rings trajectory. Magn Reson Med.

[36] Mayer D, Levin YS, Hurd RE, Glover GH, Spielman DM (2006). Fast metabolic imaging of systems with sparse spectra: Application for hyperpolarized 13C imaging. Magn Reson Med.

